# Enduring Efficacy and Clinical Outcomes of Combined Palliative Chemotherapy With Gefitinib, Methotrexate, and Cyclophosphamide in Advanced Oral Cancer: A 3.5-Year Case Study of Carcinoma in the Buccal Mucosa and Hard Palate

**DOI:** 10.7759/cureus.46661

**Published:** 2023-10-07

**Authors:** Sohilkhan R Pathan, Nirav Asarawala, Raghunandan G Chowdappa, Rushikumar Panchal, Priyanka S Srivastava, Vishal A Patel, Kruti B Sharma, Shivangi B Pandya, Meet D Patel

**Affiliations:** 1 Clinical Research Services, Bhanubhai and Madhuben Patel Cardiac Centre, Shree Krishna Hospital, Anand, IND; 2 Medical Oncology, M. S. Patel Cancer Centre, Shree Krishna Hospital and Medical Research Centre, Anand, IND; 3 Surgical Oncology, M. S. Patel Cancer Centre, Shree Krishna Hospital and Medical Research Centre, Anand, IND; 4 Radiation Oncology, M. S. Patel Cancer Centre, Shree Krishna Hospital and Medical Research Centre, Anand, IND; 5 Medicine, Pramukhswami Medical College, Anand, IND

**Keywords:** palliative chemotherapy, cancer management, case report, cyclophosphamide, methotrexate, gefitinib, multidisciplinary approach, oral squamous cell carcinoma

## Abstract

This case report outlines the diagnostic and treatment experience of a 50-year-old male diagnosed with moderately differentiated squamous cell carcinoma (SCC) in the right lower alveolus. It underscores the challenges of oral squamous cell carcinoma (OSCC) diagnosis and management, emphasizing the need for comprehensive multidisciplinary approaches. The patient's initial presentation with persistent mandibular pain highlighted the complexities of diagnosing oral and maxillofacial pathologies. A detailed clinical examination revealed unique ulceroproliferative growth, showcasing the importance of meticulous clinical assessment. Histopathological confirmation solidified the diagnosis. Treatment involved surgery, adjuvant radiotherapy, and concurrent chemotherapy. Post-chemotherapy, the patient responded positively, underlining treatment efficacy. Transitioning to oral chemotherapy demonstrated adaptability. Vigilant follow-up, exemplified by detecting non-healing ulcers and erosions, is crucial for early intervention. This case informs oral squamous cell carcinoma management. Integrated therapy's success underscores the value of combining surgery, chemotherapy, and radiotherapy. The patient's response to gefitinib, cyclophosphamide, and methotrexate suggests promise for targeted therapies. Patient-centered care, interdisciplinary collaboration, and adaptability are vital. This case report illustrates oral squamous cell carcinoma eradication through multidimensional treatment. The patient's journey highlights accurate diagnosis, adaptable therapy, and vigilant follow-up. It informs the field and fosters further research and innovation.

## Introduction

Buccal mucosa cancer primarily occurs along the occlusal plane and is characterized by pain and ulceration, which are usually accompanied by a buccal mass. Squamous cell carcinoma (SCC) of the buccal mucosa is rare and accounts for approximately 10% of all oral cancers [1]. Buccal mucosa SCC is known to grow more rapidly and penetrate well, with a higher recurrence rate than oral SCCs at other sites. Therefore, buccal mucosa SCC requires careful treatment, even in the early stages. The buccal mucosa is anatomically connected to the vestibule of the maxilla and mandible, retromolar trigone, and masseter muscle. Thus, buccal mucosa cancer can invade adjacent structures, such as upper and lower jaws, masticatory muscles, and cheeks, often rendering surgical resection and reconstruction more challenging, particularly when cancer invades the masticator space; furthermore, it is even more complicated when the mouth opening is limited. Following surgical resection of the tumor, appropriate reconstruction is necessary to minimize functional and esthetic issues [1].

Carcinoma of the buccal mucosa is the most common oral cavity cancer in India. It accounts for approximately 77,000 new cases and 52,000 deaths a year, representing a quarter of all incident cases worldwide. It is the second and fifth most common cancer among Indian men and women, respectively [2]. The relatively high incidence of oral cancer in India is mainly because of the extremely popular use of the smokeless tobacco product called gutkha and betel quid chewing (with or without tobacco), which renders its population and especially its youth at greater risk of developing oral submucous fibrosis, a premalignant disease resulting in an increased incidence of oral cancer in younger patients [3].

Moreover, hard plate cancer (HPC) is an uncommon malignant tumor. Although rare, these tumors usually have more aggressive behavior than other oral cancer sites. Etiologic factors dominated by alcohol and tobacco consumption are similar to those of other oral cavity cancers [4]. HPC represents approximately 1-3.5% of oral cavity cancers and is most often a squamous cell carcinoma [5]. A variety of treatments have been used to treat hard palate cancer, including surgery, radiotherapy, chemoradiation, and combinations of these modalities. Even rare compared with other oral cancer sites by their local aggressiveness, palate squamous cell carcinomas may cause significant disabling functional morbidities involving speech, mastication, and swallowing [6,7].

Long-term survival reflects cure and is a positive measure that can be used by planners and health professionals to discuss the outcome of cancer diagnosis and treatment. Literature on the management and survival of cancers in the West is widely available, but data in the Indian context are sparse. The few studies conducted in India have reported five-year survival rates for buccal mucosa cancers ranging from 80% for stage I disease to 5-15% for locally advanced disease [8]. The literature data show that the overall five-year survival rates of hard palate SCCs vary between 33% and 86% depending on tumor presentation and treatment modality [9,10].

## Case presentation

**Figure 1:**
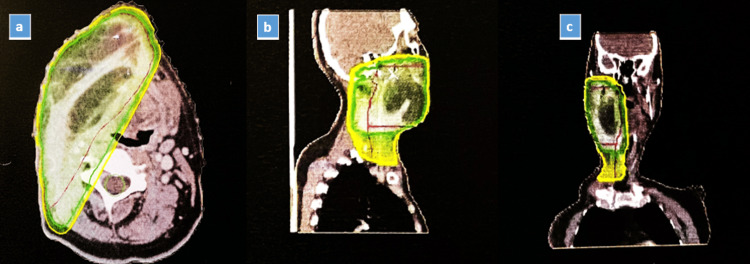
(A) Transverse, (B) sagittal, (C) coronal view of buccal mucosa radiation targeted area (64 Gy to be delivered). Gy: gray.

On day 1, a 50-year-old male presented to the ENT outpatient department with persistent right mandibular pain for six months. Clinical examination revealed a 3 cm × 3 cm ulceroproliferative growth on the right lower alveolus, confirmed as moderately differentiated squamous cell carcinoma through punch biopsies. On day 3, a CT scan showed a well-defined lesion originating from the right buccal mucosa with heterogeneous enhancement, indicative of malignancy. Enlarged cervical lymph nodes were also detected. On day 15, the patient underwent a complex surgical procedure involving right-side composite resection, hemimandibulectomy, and reconstruction with a right pectoralis major myocutaneous flap. Histopathology confirmed complete tumor removal. Post-surgery, after ICU care, the patient was discharged on the seventh postoperative day with a comprehensive post-discharge plan. Follow-ups showed a healthy flap and stable vital signs. On day 45, adjuvant treatment commenced with six cycles of weekly cisplatin and radiotherapy totaling 64 Gy over six weeks (Figure 1). This multidisciplinary approach aims to optimize the patient's prognosis by enhancing recovery and reducing disease recurrence or progression.

**Figure 2:**
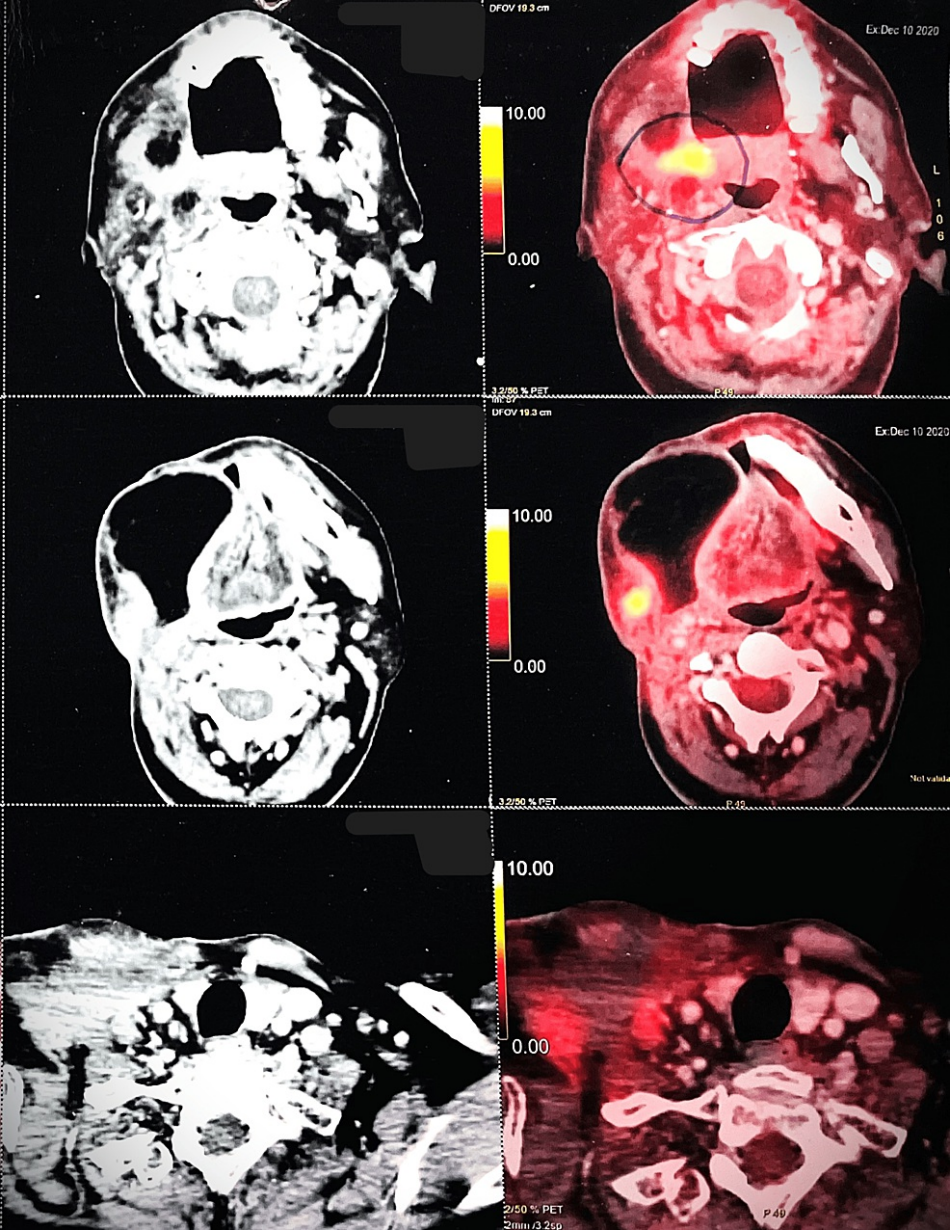
Images shows metabolically active ill-defined enhancing areas in and around flap margins at operated site - inflammatory more likely than malignant lesions.

After completing 90 days of treatment without interruptions, the patient displayed compliance and treatment efficacy, managing manageable side effects with analgesics and anesthetic gel. A detailed post-treatment plan included outpatient visits every two months in the first year, every three months in the second year, every six months from the third to the fifth year, and yearly thereafter, focusing on oral hygiene and a balanced diet for long-term well-being. Between the 90th and 300th days post-treatment, consistent outpatient attendance revealed a healthy reconstructed flap, indicating successful recovery without complications and early issue identification, reflecting the patient's dedication and treatment effectiveness. A PET CT scan (Figure 2) detected metabolic activity near the flap margins, likely due to inflammation. Ill-defined and enhanced areas were confined to the flap margins, with no signs of active loco-regional lymph nodes or distant metastasis, reassuring regarding regional and systemic health. Ongoing monitoring and clinical assessment are essential to fully understanding these inflammatory observations.

From day 300 to day 592, the patient maintained a consistently positive clinical course with no major complications or adverse events. The medical condition remained stable, showing no disease recurrence or unexpected deviations. Vigilant monitoring found no signs necessitating immediate medical attention. This prolonged stability emphasizes the patient's strong response to initial treatment and the significance of continued follow-up for comprehensive care.

**Figure 3:**
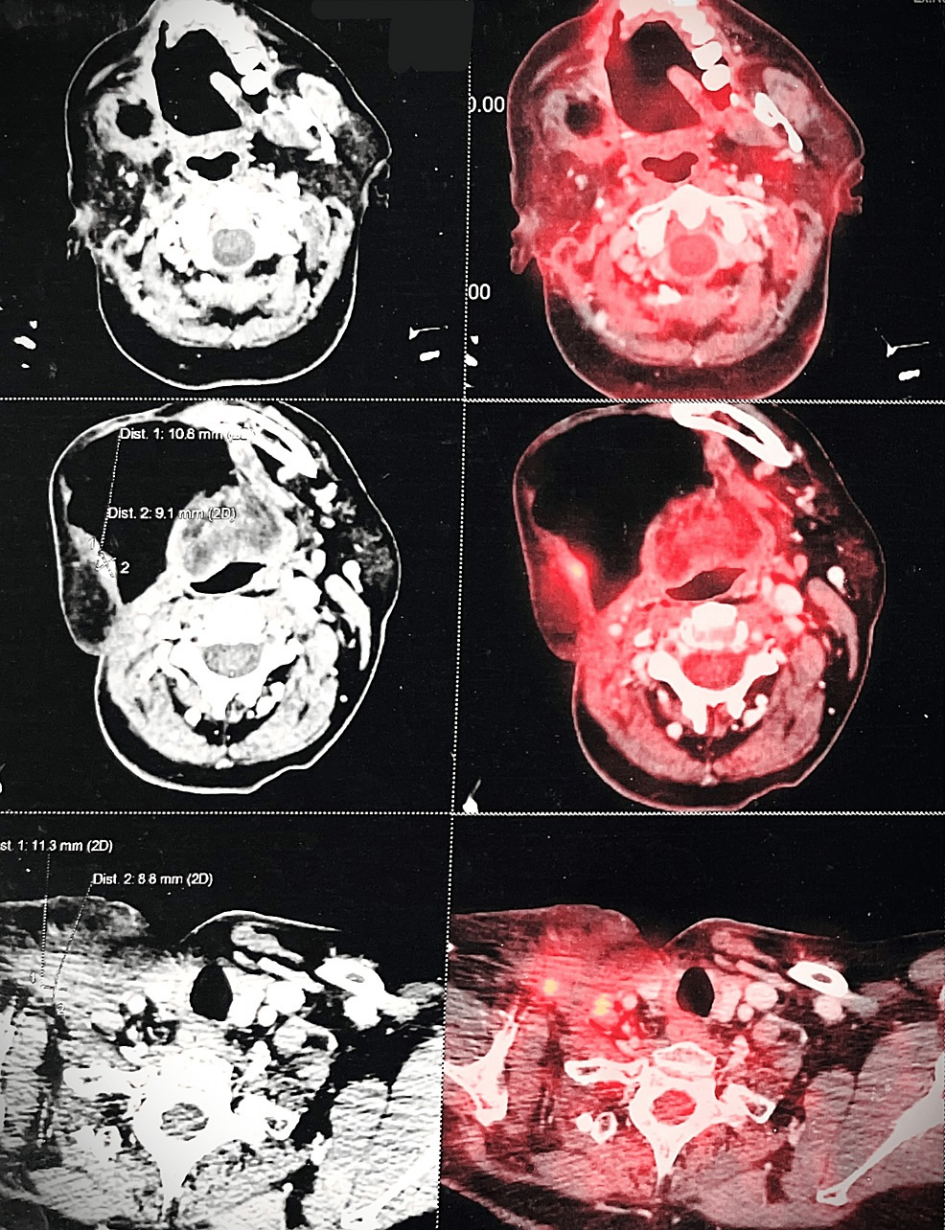
Images shows no evidence of metabolically active lesion of hard palate, as compared to previous PET-CT image shows reduction in size and metabolic activity of enhancing lesion in postero-lateral margin of flap-inflammatory. Reduction in size and metabolic activity of ill-defined enhancing lesion in right supraclavicular region - likely inflammatory.

On day 592 post-initial treatment, the patient identified a non-healing ulcer on the previously treated right hard palate, prompting a need for thorough evaluation and diagnosis. To assess the ulcer's cause and implications for post-treatment surveillance and potential recurrence, a PET CT scan was advised. The scan detected changes in size and metabolic activity of enhancing lesions in two areas, likely due to inflammation. No signs of active regional or distant metastasis were found. The patient was treated with antibiotics and steroids for the ulcer based on these findings (Figure 3). This proactive health monitoring approach fosters timely diagnosis and intervention in response to evolving health challenges.

Despite treatment with antibiotics and steroids, the patient's condition did not improve. On day 613 post-initial treatment, further evaluation was sought due to the persistent non-healing ulcer on the right hard palate. A surgeon's examination at the OPD revealed ulceroproliferative growth with specific characteristics, including ill-defined margins, everted edges, slough on the floor, tenderness, and induration around the periphery. Tongue movements were normal. These findings necessitated a punch biopsy on day 617, which confirmed the diagnosis as moderately differentiated squamous cell carcinoma, providing definitive clarity about the nature of the growth on the right hard palate.

**Figure 4:**
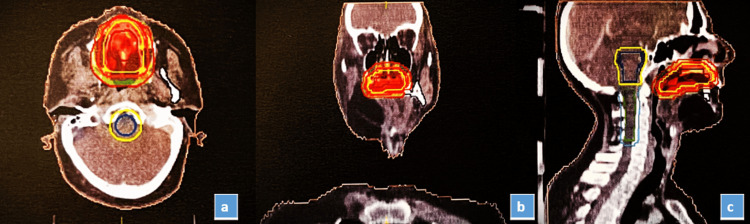
(A) Transverse, (B) coronal, (C) sagittal view of hard palate radiation targeted area (66 Gy to be delivered). Gy: gray.

From day 666 to day 729, the patient received chemotherapy with paclitaxel (290 mg) and carboplatin (450 mg) every 21 days, accompanied by Inj GCSF (granulocyte colony-stimulating factor) at 300 mcg subcutaneously for three consecutive days during each cycle. On day 732, following three chemotherapy cycles, a CT scan showed a significant reduction in the size of the malignant mass on the left side of the hard palate, indicating a positive response to chemotherapy. On day 735, radiotherapy began, with a total dose of 66 Gy delivered over 30 fractions in six weeks, using volumetric modulated arc therapy (VMAT) for precise targeting of the GTV_Palate (gross tumor volume) (Figure 4). This marks a pivotal step in the patient's treatment journey, emphasizing the commitment to achieve the best possible outcome and long-term recovery.

**Figure 5:**
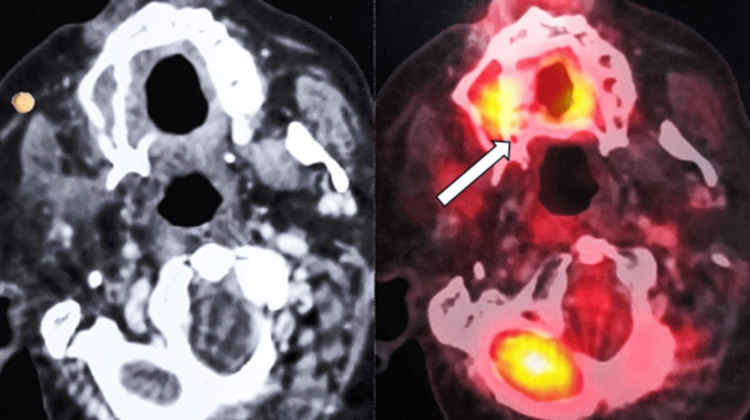
18F-FDG PET/CT scan with contrast. FDG avid enhancing lesion in left side of hard palate causes focal erosion of hard palate and adjacent floor of nasal bone, measures approx. 13 mm × 10 mm × 15 mm, SUVmax: 7.2 - shows residual malignant lesion. FDG avid enhancing lesion seen in right side of hard palate, adjacent right upper alveolar region causes erosion of floor of maxillary sinus with minimal intrasinus extension, lesion measures approx. 17 mm × 7 mm × 25 mm, SUVmax: 7.1 - shows residual malignant lesion. FDG: fluorodeoxyglucose; PET: positron emission tomography.

On day 839, a PET CT scan assessed the patient's response to treatment. Notably, the lesion in the postero-lateral margin of the flap was fully resolved, signifying successful eradication. However, the scan identified metabolically active and enhancing lesions on the left and right sides of the hard palate, indicating residual malignancy in these regions. Encouragingly, no active loco-regional lymph nodes or distant metastasis were found, reassuring regarding cancer spread. Moreover, no definite metastatic lesions were identified at critical, distant sites. While some areas are resolved completely, the presence of active lesions in specific hard palate regions suggests persistent malignancy. Nevertheless, the absence of lymph node involvement and distant metastasis supports continued treatment and management for optimal outcomes (Figure 5).

On day 840, the patient transitioned to oral palliative chemotherapy due to the absence of complete cancer resolution after three chemotherapy cycles and 32 concurrent radiation therapy sessions. The regimen includes daily gefitinib (250 mg), weekly methotrexate (2.5 mg), and cyclophosphamide (500 mg) as prescribed. Structured follow-up assessments after each treatment cycle evaluate disease progression and treatment effectiveness. On day 930, a CT scan revealed erosions in the central area of the hard palate, suggesting potential bony destruction due to the malignancy. The left cervical lymph nodes exhibited homogeneous enhancement, likely due to a reactive cause. These findings emphasize the need for close monitoring and clinical evaluation, especially regarding the lymph nodes.

**Figure 6:**
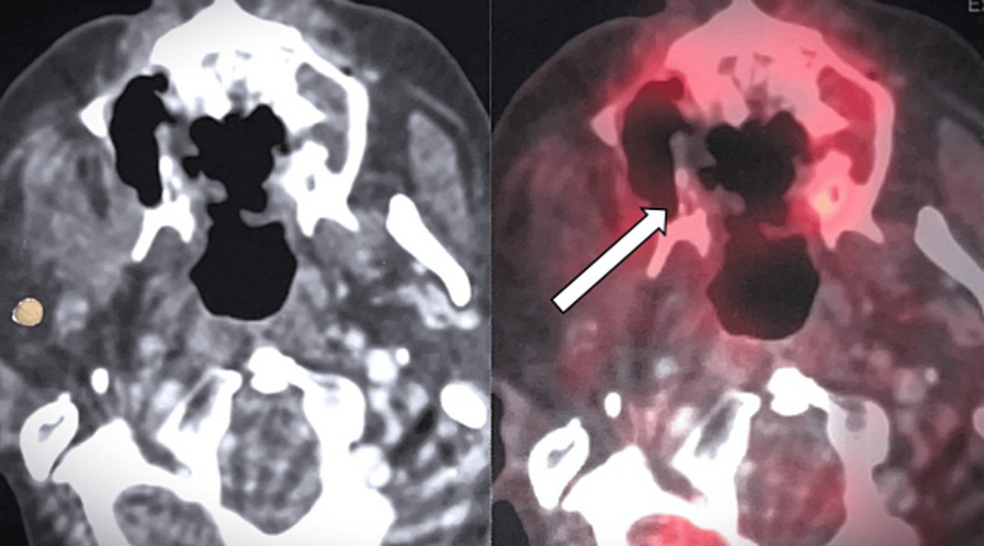
18F-FDG PET/CT scan with contrast. No evidence of FDG avid enhancing lesion seen in left side of hard palate with post-treatment defect noted (previously 13 mm × 10 mm × 15 mm, SUVmax: 7.2). No evidence of FDG avid-enhancing lesion seen in right side of hard palate, adjacent right upper alveolar region with post-treatment defect noted (previously 17 mm × 7 mm × 25 mm, SUVmax: 7.1). FDG: fluorodeoxyglucose, PET: positron emission tomography.

After extending the patient's treatment by three cycles with the same regimen, a day 1000 PET CT scan showed post-operative changes in the oral cavity and neck. Importantly, there were no FDG-avid-enhancing lesions on both sides of the hard palate, signifying resolution. The previously identified lesion in the flap's margin was no longer observed. Left cervical lymph nodes in levels II, III, and IV showed non-to-low-grade FDG avidity, suggesting a reactive nature. No FDG-avid focal lesions indicative of metastasis were found in the lungs, liver, bones, or elsewhere in the body. These results suggest a positive response to the extended palliative chemotherapy, with improvements in treated regions and no evidence of metastasis (Figure 6).

After a thorough evaluation on day 1000, we have reached a highly promising conclusion regarding the combined impact of gefitinib, methotrexate, and cyclophosphamide on the patient's cancer treatment. This treatment journey has yielded remarkable results, with the disappearance of cancerous lesions on both sides of the hard palate. This positive response reflects the dedication of both the medical team and the patient in the fight against cancer. Regular follow-up and monitoring will be crucial to assessing the durability of these outcomes. Combining targeted therapy with traditional chemotherapy agents, this comprehensive approach played a crucial role in achieving the patient's positive treatment response. It underscores the importance of tailored treatment plans that leverage different medication mechanisms to achieve the best outcomes for cancer patients. The success of this case highlights several key aspects of effective cancer treatment, including collaborative efforts between the medical team and the patient, personalized treatment approaches, regular monitoring, and the potential for innovative therapies to reshape cancer treatment paradigms. The medical team's commitment to excellence, innovation, and compassionate care remains unwavering as the patient's journey continues, with the goal of achieving the best possible medical outcome.

## Discussion

Our case report demonstrates a positive response to a combined treatment regimen of gefitinib, methotrexate, and cyclophosphamide in oral squamous cell carcinoma (OSCC) treatment. This contrasts with a study by Irshad et al., which showed no partial response to methotrexate [11]. Additionally, a study by Kushwaha et al. on recurrent head and neck squamous cell carcinoma found no significant differences in overall survival or objective response rates among gefitinib, methotrexate, and methotrexate plus 5-fluorouracil (5-FU). However, the study noted an improvement in quality of life with gefitinib, highlighting the complex and context-specific nature of cancer treatment outcomes [12].

In our comprehensive case report, we underscore the pivotal role of precise diagnostics in initiating effective treatment strategies. The synergy between clinical assessments and histopathological confirmation, leading to the diagnosis of moderately differentiated squamous cell carcinoma, highlights the paramount importance of accurate diagnostics.

Our patient's journey through a multimodal treatment approach, including surgery, adjuvant radiotherapy, and concurrent chemotherapy, showcases the effectiveness of combined therapies in achieving tumor eradication. The subsequent transition to oral chemotherapy exemplifies a patient-centered and adaptive treatment approach. The remarkable absence of lesions post-treatment attests to the potential of targeted therapies in achieving disease regression. Throughout this journey, the case emphasizes the critical importance of meticulous follow-up and surveillance, enabling timely intervention.

In summary, our case report provides insights into effective OSCC management, emphasizing the significance of accurate diagnosis, multidisciplinary treatment, patient-centered care, and vigilant surveillance in achieving positive outcomes.

## Conclusions

This comprehensive case report concludes with the remarkable achievement of OSCC management, showcasing a precision-driven, adaptive approach and vigilant post-treatment surveillance. The journey emphasizes the pivotal role of precise diagnostics in tailoring treatments and underscores the enduring commitment to the patient's well-being.

The multidisciplinary approach, encompassing surgery, radiotherapy, and chemotherapy, demonstrates the effectiveness of combined therapies in achieving tumor eradication. Notably, the transition to oral chemotherapy with gefitinib, methotrexate, and cyclophosphamide attains disease regression, highlighting the potential of targeted molecular treatments. Crucially, the patient's positive response to palliative chemotherapy underscores its efficacy in achieving the best results. This case carries broad implications, affirming the significance of patient-centric care, interdisciplinary collaboration, and adaptable treatment strategies. In summary, it represents a beacon of hope and innovation in OSCC management, shaping future practices and emphasizing the profound impact of palliative chemotherapy on achieving optimal outcomes.
